# Concomitant trauma of brain and upper cervical spine: lessons in injury patterns and outcomes

**DOI:** 10.1007/s00068-023-02278-w

**Published:** 2023-05-15

**Authors:** Nicolò Marchesini, Andreas K. Demetriades, Wilco C. Peul, Nicola Tommasi, Paolo Zanatta, Giampietro Pinna, Francesco Sala

**Affiliations:** 1 Department of Neurosurgery, University Hospital Borgo Trento, Verona, Italy; 2 Department of Neurosurgery, Royal Infirmary, Edinburgh, UK; 3 University Neurosurgical Center Holland, HMC-HAGA The Hague & LUMC, University of Leiden, Leiden, The Netherlands; 4 Centre of Economic Documentation (CIDE), University of Verona, Verona, Italy; 5 Department of Neurocritical Care, University Hospital Borgo Trento, Verona, Italy

**Keywords:** Concomitant craniospinal injury, Traumatic brain injury, Traumatic cervical spinal injury, Upper cervical spine trauma

## Abstract

**Purpose:**

The literature on concomitant traumatic brain injury (TBI) and traumatic spinal injury is sparse and a few, if any, studies focus on concomitant TBI and associated upper cervical injury. The objective of this study was to fill this gap and to define demographics, patterns of injury, and clinical data of this specific population.

**Methods:**

Records of patients admitted at a single trauma centre with the main diagnosis of TBI and concomitant C0–C1–C2 injury (upper cervical spine) were identified and reviewed. Demographics, clinical, and radiological variables were analyzed and compared to those of patients with TBI and: (i) C3–C7 injury (lower cervical spine); (ii) any other part of the spine other than C1–C2 injury (non-upper cervical); (iii) T1–L5 injury (thoracolumbar).

**Results:**

1545 patients were admitted with TBI and an associated C1–C2 injury was found in 22 (1.4%). The mean age was 64 years, and 54.5% were females. Females had a higher rate of concomitant upper cervical injury (*p = *0.046 vs non-upper cervical; *p = *0.050 vs thoracolumbar). Patients with an upper cervical injury were significantly older (*p = *0.034 vs lower cervical; *p = *0.030 vs non-upper cervical). Patients older than 55 years old had higher odds of an upper cervical injury when compared to the other groups (OR = 2.75). The main mechanism of trauma was road accidents (RAs) (10/22; 45.5%) All pedestrian injuries occurred in the upper cervical injured group (*p = *0.015). ICU length of stay was longer for patients with an upper cervical injury (*p = *0.018). Four patients died in the upper cervical injury group (18.2%), and no death occurred in other comparator groups (*p = *0.003).

**Conclusions:**

The rate of concomitant cranial and upper cervical spine injury was 1.4%. Risk factors were female gender, age ≥ 55, and pedestrians. RAs were the most common mechanism of injury. There was an association between the upper cervical injury group and longer ICU stay as well as higher mortality rates. Increased understanding of the pattern of concomitant craniospinal injury can help guide comprehensive diagnosis, avoid missed injuries, and appropriate treatment.

## Introduction

Traumatic Brain Injury **(**TBI) can be defined as the result of an insult to the brain caused by the application of a “bump, blow or jolt to the head” which can, in turn, result in fractures, bleeding, and/or altered brain functioning [1, 2]. TBI and cervical spine injury, however, may occur together.

Traumatic spinal injury consists of a variety of damages to the spinal cord and/or to the bony elements of the spinal column that can determine mechanical instability, pain, impaired mobility, and various grades of neurological deficit [3]. Both conditions may carry a significant proportion of mortality and morbidity [1, 4]. The neurological damage itself and the associated complications that may arise can be responsible for high-impact social, economic, and healthcare costs [5–9]. Indeed, such patients can often suffer multi-system injury or dysfunction [10].

Due to the anatomical and biomechanical relationship between the cranium and the cervical spine, a concomitant craniocervical injury may occur and, due to the possible serious consequences of a missed injury, is necessary to exclude [11]. Associated cervical injury in TBI has a varied reported incidence; however, an increasing prevalence of spinal trauma in TBI has been observed, with indeed a fourfold increase in cervical per se spine fractures [12]. A recent systematic review with meta-analysis reported that, in patients with TBI, the prevalence of concomitant cervical spinal injury is 6.5%; and this rises to 11.7% in cases of motor vehicle accidents [2].

While several studies have examined the epidemiology, pathophysiology, and clinical course of concomitant traumatic brain and spinal injury patients, a few—if any—studies with high granularity have specifically focused on concomitant craniospinal injury with a craniovertebral junction or upper cervical segment involvement [13–22]. As this part of the spinal column has particular anatomical and biomechanical features, and as injuries to the upper cervical spine are potentially devastating, we believed that it was worthwhile to analyze this sub-population [23, 24].

The aim and objective of the present study was to evaluate the presence of concomitant cranial and upper cervical spine injury; to identify any potential patterns of injury, whether musculoskeletal and/or neurological; and to observe any potential particularities in the investigation, treatment, and prognosis of such cases. Comparing the findings of this specific group to patients with concomitant TBI and spinal injuries in other vertebral segments, we aim to unshadow any similarities or differences between these populations.

## Methods

This was a single-centre retrospective cohort study of patients treated in a level 1 trauma centre as specified by the regional law and located in Verona, a province in the northeastern part of Italy [25]. The neurosurgery service is the only one in the province and provides 24-h urgent care to a population of nearly 930.000 inhabitants and around additional 5 million tourist arrivals each year according to the pre-pandemic data [26]. Trauma victims can reach neurosurgical consultation either after a direct presentation to this hospital or after teleconsultation from one of five hospitals equipped with an Emergency Department (ED) and CT scans but without on-site neurosurgical service. In both cases, patients are triaged and evaluated in the ED and, according to internationally recognized clinical criteria and national guidelines, they are screened by the ED physician for head and spinal injury [27, 28]. Patients with mild TBI are not *routinely* screened for spinal trauma and, as a general principle, when head CT scan and spinal imaging (X-ray, CT) are normal, neurosurgical consultation is not requested. Consequently, those patients are not hospitalized in the Neurosurgical ward nor the Neurological Intensive Care Unit^25^. Vice versa, all major traumas are investigated by a whole-body CT, and based on imaging and clinical findings, neurosurgical consultation might be requested.

A prospective registry for TBI and spinal trauma with granular information has not been implemented yet. Thus, data for this study were obtained by a retrospective review of the medical records. Included patients were admitted to the Neurosurgical ward or Neurological Intensive Care Unit between January 2013 and December 2020 with the diagnosis of TBI as the main diagnostic code. Associated/concomitant spinal injuries were identified with the use of institutional diagnostic coding.

**Fig. 1:**
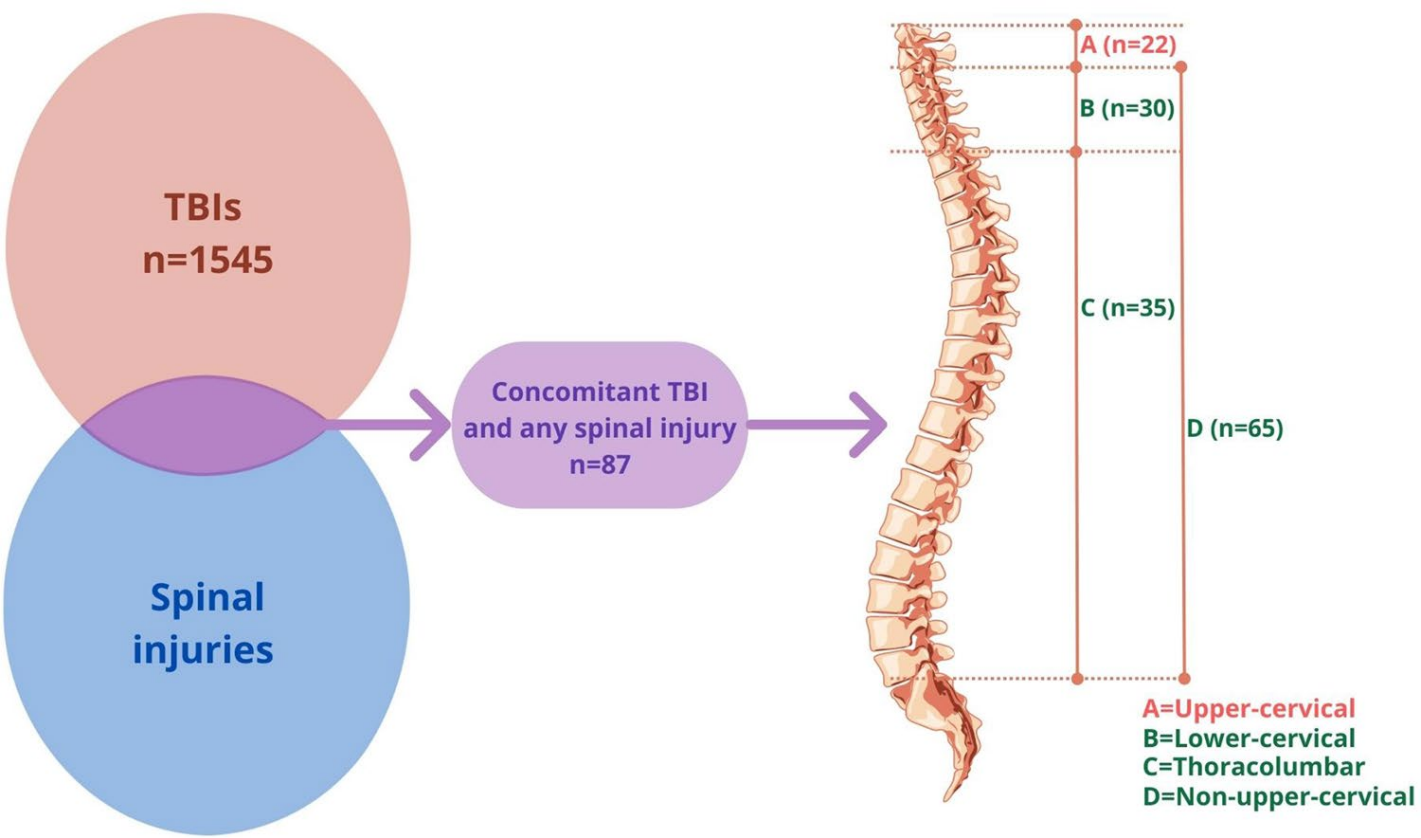
The figure shows the groups considered in the study. Of 1545 patients with TBI, 87 had a concomitant spinal injury in any part of the spinal column. Of these, 22 (group A) had an upper cervical injury. Comparators were those with TBI *plus* a lower cervical injury (30 cases, group B), a thoracolumbar injury (35 cases, group C), and a non-upper cervical injury (65 cases, group D)

Inclusion criteria were TBI of any severity on arrival to the hospital, warranting hospitalization in the neurosurgical ward or the neuro-ICU and including any of the following findings correlating “positive” CT scan: vault or base skull fracture, epidural hematoma, subdural hematoma, subarachnoid hemorrhage, and brain contusion. Patients with associated spinal injury at any level and of any type were selected from those with a TBI as defined above. Finally, patients with involvement of C0–C1–C2 were included for specific analysis (upper cervical spine group). Comparisons were performed between the upper cervical spine group and patients with a C3–C7 injury (lower cervical spine group); patients with an injury in any other part than C1–C2 (non-upper cervical injury group); and patients with a T1–L5 injury (thoracolumbar injury group) (Fig. 1). A spinal injury was defined as a fracture and/or a ligamentous injury as detected by CT and/or MRI and severe enough to require treatment (collar, halo-vest, surgery). Cases with associated injuries in body regions other than the head or the spine were identified [29].

Demographic (gender, age), clinical (GCS score after resuscitation, ICU requirement, neurological deficit, associated injuries, need for cranial and spinal surgery, mortality, and length of hospital stay), and radiological (C1 and C2 injury features, and head CT finding) variables were analyzed by descriptive and inferential statistics. The mechanism of trauma was classified as per road accidents (RAs = any accident occurred on a road: car, pedestrian, motorcycle, and bicycle); fall from standing height; precipitation (i.e., greater than one’s height); other (hit by an object; sport; and aggression). Due to the retrospective nature of the study and the quality of data collection, reliable clinical information was available till discharge.

The difference between the means for continuous variables was assessed using the *t* test. Pearson’s *χ*^2^ test was used to assess measures of association in frequency tables. Univariate analysis was used to identify potential risk factors. Variables with higher significance were subsequently included in multivariate analysis. Variables and two-way interaction terms were subjected to multivariate analysis using logistic regression model. Odds ratio were presented as an odds ratio (OR) and a 95% Confidence Interval. Statistical analysis was performed using a commercially available software (Stata version 16.1). For statistical significance, values of *p* < 0.05 were considered.

Ethics approval was not required for this study.

## Results

### Injury demographics

In the examined period, 1545 patients were admitted with a diagnosis of TBI and with a “positive” head CT scan. Of these, 87 (5.6%) had any spine injury and 52 (3.4%) had an associated cervical injury confirmed by CT w/o MRI. From this group of 87 concomitant spinal injuries, 22/1545 only (1.4%) were found to have a C1–C2 injury (Table 1). Ten were males (45.5%) and 12 females (54.5%) with a mean age of 64 years (range 18–90; median 71; SD 20.7).

**Table 1:** Details of the 22 patients with TBI and associated C1–C2 trauma (upper cervical group)

Mechanism	GCS on the scene and GOS at discharge	Head CT	C1 injury features	C2 injury features	Other vertebral levels involved	Associated injuries	Operations
MVA (P)	14–3	Sa, c	Anterior arch	–	–	LL, tx	–
MVA (B)	3–1	Sa, c	C0–C1 subluxation	Body	–	UL, tx, Abd	General surgery
MVA (C)	14–4	sa	–	Articular	CT, Th, TL	tx, pelv	–
Precipitation	3–1	sa	Posterior arch	–	C, CT	LL, mx, tx	Orthopedics, lower cervical
Fall	8–1	v, sd,	Anterior + posterior arch	–	C, CT	mx	–
Fall	15–3	sd, sa, c	–	Anderson 2	–	–	–
Fall	15–5	v, sd,	–	Body	–	–	–
MVA (P)	11–4	sa,c	Subluxation	–	–	–	–
Object	15–5	sd,	–	Anderson 2	–	–	–
MVA (P)	15–5	sa,	Anterior arch, lateral mass	–	–	–	–
MVA (B)	15–4	sd	Posterior arch	Anderson 2	–	mx	–
MVA (M)	15–5	c	Posterior arch	Hangman	C, CT, Th	cv, tx	–
Fall	15–5	sd	–	Anderson 3	–	–	–
Fall	14–1	sd	–	Anderson 2	–	UL	–
Precipitation	15–5	v	–	Body, dens, articular	C	UL, mx, tx	–
MVA (M)	15–5	c	–	Body	C, CT, TL	tx	–
MVA (C)	15–5	sa	–	Transverse process	C	tx	–
Fall	15–5	v + b, sd	Lateral mass	–	–	–	–
Fall	14–4	sd	Posterior arch	–	–	–	Cranial
Precipitation	6–5	sd, sa,c	Jefferson	Body	C, CT	Mx, tx	–
Fall	13–3	v + b, sd, sa, c	–	Articular	–	–	–
MVA (C)	5–3	sd, sa, c	–	Anderson 3 + articular	Th	LL, tx, abd. pelv	Orthopedics

*RA* road accident, *P* pedestrian, *B* bike, *C* car, *T* intubated, *sa* subarachnoid hemorrhage, subdural hematoma, *c* contusion, *v* vault fracture, *b* skull base fracture, *C* cervical (C3-C6), *CT* cervico-thoracic (C7-T1), *Th* thoracic (T2-T10), *TL* thoracolumbar, *LL* lower limb injury, *UL* upper limb injury, *tx* thoracic injury, *abd* abdominal injury, *pelv* pelvic injury, *mx* maxillofacial injury, *cv* cervical injury

Thirty patients (30/1545, i.e., 1.9%) had a lower cervical spine injury (i.e., C3–C7). This means that a total of 65/1545 (4%) patients had any spinal injury other than a C1–C2 injury and 35/1545 (2.2%) had a thoracolumbar injury (without a cervical involvement). Females were 12 (54.4%) in the upper cervical injured group vs 20 (30.8%) in the non-upper cervical (*p = *0.046) and vs 10 (28.6%) in the thoracolumbar group (*p = *0.050) (Table 2). Females had significantly higher odds of upper cervical injury (Table 3). Patients with an upper cervical injury were on average older than the other groups (upper cervical: lower cervical *p = *0.034; upper cervical: non-upper cervical *p = *0.030). On univariate analysis, patients older than 55 years old had higher odds of upper cervical injury when compared to the others (OR = 2.75) 95% CI [0.95–7.9] (Table 3). However, this was not confirmed in multivariate analysis (see Table 4).

**Table 2:** Demographic and clinical characteristics of patients with C1–C2 injuries (upper cervical), C3–C7 injuries (lower cervical), C3–L5 injury (non-upper cervical) and D1–L5 injury (thoracolumbar)

	Upper cervical injuries (*n* = 22) *n* (%)	Lower cervical injuries (*n* = 30) *n* (%)	Non-upper cervical injuries (*n* = 65) *n* (%)	Thoraco-lumbar injuries (*n* = 35) *n* (%)	*p* value upper cervical vs lower cervical injuries	*p* value upper cervical vs non-upper cervical	*p* value upper cervical vs thoracolumbar injuries
Gender					0.126	**0.046**	**0.050**
Males	10 (45.5)	20 (66.7)	45 (69.2)	25 (71.4)			
Females	12 (54.5)	10 (33.3)	20 (30.8)	10 (28.6)			
Age (years)	64 ± 20,71 (18–90)	52 ± 18.4 (21–89)	54 ± 19.8 (16–89)	55 ± 21.1 (16–94)	**0.034**	**0.030**	0.076
Mechanism of injury							
RA total	10 (45.5)	13 (43.3)	31 (47.7)	18 (51.4)	0.879	0.527	0.665
Pedestrian	3 (13.6)	0 (0)	0 (0)	0 (0)	0.070*	0.015*	0.053*
Bicycle	2 (9.1)	2 (6.7)	8 (12.3)	6 (17.1)	0.569	0.512	0.331
Car	3 (13.6)	10 (33.3)	20 (30.8)	7 (20)	0.193	0.163	0.193
Motorcycle	2 (9.1)	1 (3.3)	3 (4.6)	2 (5.7)	0.383	0.373	0.503
Falls	8 (36.4)	8 (26.7)	14 (21.5)	6 (17.1)	0.327	0.137	0.093
Precipitation	3 (13.6)	4 (13.3)	12 (18.5)	8 (22.9)	0.641	0.439	0.309
Other	1 (4.5)	5 (16.6)	4 (6.2)	1 (2.9)	0.183	0.279	0.497
Neurological deficit	0 (0)	4 (13.3)	4 (6.1)	0 (0)			
GCS							
≤ 8	5 (22.7)	5 (16.7)	17 (26.2)	12 (34.3)	0.420	0.495	0.266
9–12	1 (4.5)	3 (20)	6 (9.2)	3 (8.5)	0.431	0.430	0.497
13–15	16 (72.7)	22 (73.3)	42 (64.6)	20 (57.1)	0.602	0.336	0.183
ICU requirement	10 (45.5)	12 (40)	34 (52.3)	22 (62.9)	0.456	0.379	0.155
ICU length of stay	20.5 (4–57)	8.5 (2–29)	9.9 (1–32)	10,7 (1–32)	0.068	**0.018**	0.059
Head CT findings							
Skull fracture (all)	5 (22.7)	13 (44.3)	24 (36.9)	11 (31.4)	0.105	0.169	0.345
Vault fracture	5 (22.7)	11 (36.7)	18 (27.7)	7 (20)	0.211	0.439	0.529
Skull base fracture	2 (9.1)	4 (13.3)	9 (13.8)	5 (14.3)	0.494	0.436	0.444
Epidural hematoma	0 (0)	3 (10)	4 (6.2)	1 (2.9)	0.184*	0.304*	0.614*
Subdural hematoma	12 (54.5)	15 (50)	31 (47.7)	16 (45.7)	0.746	0.578	0.516
Brain contusion	9 (40.9)	18 (60)	35 (53.8)	17 (48.6)	0.173	0.294	0.572
Subarachnoid hemorrhage	11 (50)	15 (50)	32 (49.2)	17 (48.6)	1.000	0.950	0.916
Vertebral segments							
Upper cervical	22 (100)	0 (0)	0 (0)	0 (0)	–	–	–
Lower cervical (C3–C7)	7 (31.8)	30 (100)	19 (29.2)	0(0)	–	–	–
Cervico-thoracic (C7-T1)	6 (27.3)	18 (60)	19 (29.2)	1-D1 (2.9)	–	–	–
Thoracic (T2-T10)	3 (13.6)	11 (36.7)	30 (46.2)	19 (54.3)	0.064	–	–
Thoraco-lumbar (T11-L1)	2 (9.1)	6 (20)	24 (36.9)	18 (51.4)	0.281	–	–
Lumbar (L2-L5)	0 (0)	6 (20)	17 (26.2)	11 (31.4)	0.025*	–	–
Associated injuries							
Associated injuries (all)	13 (59.1)	21 (70)	48 (73.8)	27 (77.1)	0.414	0.191	0.147
Upper limbs	3 (13.6)	4 (13.3)	6 (9.2)	2 (5.7)	0.641	0.408	0.286
Lower limbs	3 (13.6)	4 (13.3)	7 (10.8)	3 (8.6)	0.641	0.488	0.425
Maxillo-facial	5 (22.7)	12 (40)	26 (40)	14 (40)	0.156	0.113	0.145
Thoracic	10 (45.5)	13 (43.3)	36 (55.4)	23 (65.7)	0.879	0.420	0.132
Abdominal	2 (9.1)	5 (16.7)	12 (18.5)	7 (20)	0.359	0.250	0.238
Pelvic	2 (9.1)	1 (3.3)	6 (9.2)	5 (14.3)	0.379	0.984	0.695
Cranial surgery	1 (4.5)	4 (13.3)	7 (10.8)	3 (8.6)	–	–	–
Spinal surgery	1 (4.5)	9 (30)	13 (20)	4 (11.4)	–	–	–
In-hospital deaths	4 (18.2)	0 (0)	0 (0)	0 (0)	0.015*	0.003*	0.019*
GCS at discharge	14,61 (9–15)	14,53 (5–15)	14,43 (5–15)	14,34 (10–15)	0.664	0.879	0.503
Length of hospital stay	16,45 (4–57)	14,03 (2–57)	15,29 (2–114)	16,37 (2–114)	0.717	0.699	0.742

Differences between groups in the rate of each variable are reported in the last three columns. Statistically significant results are highlighted in bold

^*^Indicates a statistical analysis performed in the presence of a value “0” in one of the groups

**Table 3:** Univariate analysis of the association between the potential risk factor and upper cervical injury

	Upper cervical injury		
	Odds ratio	95% CI	*p*
Gender (base female)			
Male	0.37	(0.13–0.99)	**0.049**
Age (base < 55)			
≥ 55	2.75	(0.95–7.9)	0.061
Injury mechanism (base fall)			
Precipitation	0.43	(0.09–2.02)	0.291
RA (total)	0.56	(0.18–1.73)	0.319
Other	0.21	(0.02–2.08)	0.186
GCS (base 14–15)			
9–13	0.43	(0.04–3.92)	0.460
3–8	0.77	(0.24–2.44)	0.660
Cranial fracture-generic (base no)			
Yes	0.50	(0.16–1.53)	0.227
Vault fracture (base no)			
Yes	0.76	(0.24–2.39)	0.649
Cranial base fracture (base no)			
Yes	0.62	(0.12–3.12)	0.565
Epidural hematoma (base no)			
Yes	–	–	–
Subdural hematoma (base no)			
Yes	1.31	(0.49–3.47)	0.579
Subarachnoid hemorrhage (base no)			
Yes	1.03	(0.39–2.7)	0.950
Contusion (base no)			
Yes	0.59	(0.22–1.58)	0.297
Associated injury-any (base no)			
Yes	0.51	(0.18–1.41)	0.195
Upper limb injury (base no)			
Yes	1.55	(0.35–6.8)	0.560
Lower limb injury (base no)			
Yes	1.30	(0.30–5.56)	0.716
Maxillo-facial injury (base no)			
Yes	0.44	(0.14–1.34)	0.150
Thoracic injury (base no)			
Yes	0.67	(0.25–1.77)	0.421
Abdominal injury (base no)			
Yes	0.44	(0.09–2.15)	0.312

Statistically significant results are highlighted in bold

**Table 4:** Multivariate analysis of the association between potential risk factors and upper cervical injury

	Upper cervical injury		
	Odds ratio	95% CI	*p*
Gender (base female)			
Male	0.43	(0.14–1.26)	0.125
Age (base < 55)			
≥ 55	1.98	(0.55–7.05)	0.290
Injury mechanism (base fall)			
Precipitation	0.82	(0.12–5.47)	0.843
RA (total)	0.97	(0.21–4.42)	0.977
Other	0.35	(0.02–4.26)	0.412
GCS (base 3–8)			
9–15	0.96	(0.26–3.54)	0.954
Vault fracture (base no)			
Yes	0.63	(0.17–2.26)	0.483
Cranial base fracture (base no)			
Yes	0.91	(0.15–5.25)	0.917

Potential risk factors were chosen among those showing higher significance in univariate analysis

In the upper cervical injury group, the main mechanism of trauma was RAs (10/22; 45.5%). However, this was the group with the lowest rate of car accidents and the one with the highest rate of motorcycle accidents (*p >* 0.05; Table 2). All three pedestrian injuries of the whole series occurred in this group. Bicycle accidents were more common in the thoracolumbar group (*p >* 0.05). The second most common cause of upper cervical injury was falls (8/22, 36.4%), with a progressively decreasing rate for this mechanism in the craniocaudal direction (upper cervical: lower cervical: non-upper cervical: thoracolumbar = 36.4%: 26.7%: 21.5%: 17.1%; *p >* 0.05). The mechanism of injury was not found to be statistically significant for upper cervical injury in univariate analysis and in multivariate analysis (Table 3).

### Characteristics of brain injury

The most common findings in head CT scans of the upper cervical spine injury group were subdural hematoma and brain contusions (12; 54.5% and 9; 40.9%). In the upper cervical injury group, TBI was most commonly mild (16; 72.7%). The severity of head injury did not reach statistical significance as regards its association with upper cervical or lower cervical or thoracolumbar spinal column injury (Tables 2 and 3). The same stands for the GCS at the time of discharge.

Subdural hematomas were more frequent in upper cervical spine injuries than in the other groups, while the opposite occurred for brain contusions (statistically nonsignificant). Traumatic subarachnoid hemorrhage was more equally distributed among all groups. No patient with upper cervical injury presented with an epidural hematoma, while this finding was present in 10% of the lower cervical injury group (*p >* 0.05). The rate of skull fractures was lower in the upper cervical injury group than in the other groups, and this difference was more evident in vault fractures than skull base fractures (*p >* 0.05).

Evacuation of an acute subdural hematoma was required in one patient under treatment with aspirin and Clopidogrel who had an associated posterior C1 arch fracture. The clinical course was favourable and the patient was discharged to a rehabilitation facility with a GOS (Glasgow Outcome Scale) of 4. Univariate analysis did not reveal any statistical significance for any of the head CT scan findings (Table 3).

### Characteristics and management of upper cervical spine injuries

Seven patients (7/22, 31.8%) had an isolated C1 injury and 11/22 had an isolated C2 injury (50%), whereas 4/22 had both C1 and C2 involvement (18.2%). Of note, this series did not contain any occipital condyle fractures.

None of the upper cervical spine injuries did have a spinal cord injury (SCI) attributable to C1 or C2 injuries. However, one severe injured patient with a C1 posterior arch fracture had an associated C6–C7 spinal cord contusion and was operated on by a C6–C7 anterior cervical discectomy and fusion. The clinical course was complicated by sepsis and the patient died during his ICU stay. Additionally, one patient was found to have a C0–C1 subluxation at the CT. The clinical course was rapidly fatal and an MRI to confirm spinal cord damage was not obtained.

One case of vertebral artery dissection occurred in a patient in their late teens with a posterior C1 arch and Hangman fractures. Conservative treatment was adopted (both for the fractures and dissection) and the patient was discharged autonomous at home.

Thirteen upper cervical injuries (59.1%) were detected during a whole-body polytrauma CT protocol, while the remaining were selectively investigated by a cervical CT scan. Eleven patients (50%) underwent cervical MRI.

Among the observed C1 injuries, there were: one isolated anterior arch fracture, four isolated posterior arch fractures, one anterior plus posterior arch fracture; one anterior arch fracture with lateral mass involvement, one C1/2 ligamentous subluxation, one isolated lateral mass fracture, and one Jefferson fracture. Additionally, one case of C0–C1 subluxation was also encountered.

Among the C2 fractures, there were four Anderson II and two Anderson III odontoid fractures, four pure body fractures, two articular fractures, one transverse process, and one mixed and one Hangman’s fracture.

All patients with a concomitant craniospinal injury that involved the upper cervical spine were treated conservatively with a cervical collar as regards the upper cervical spine injury. The single patient with posterior C1 arch and Hangman’s fractures was treated by placement of a Halo ring and vest.

### Associated injuries in body regions other than the head or spine

Thirteen patients (59.1%) had an associated injury in body parts other than the head or the spinal column and the most common was the thorax, (10/22; 45.5%). Overall, in the upper cervical spine group, the number of patients with associated injuries was inferior to that in the other groups. (*p >* 0.05). The rate of maxillofacial, thoracic, abdominal, and pelvic injuries was inferior in the upper cervical injury group than in the thoracolumbar injury group. Limb injuries were more common in the upper cervical injury group (*p >* 0.05). The rate of limb and thoracic injuries was similar between the upper cervical and lower cervical injury groups (*p >* 0.05). Univariate and multivariate analyses showed no statistical significance for any associated injury with an upper cervical spine injury.

In the upper cervical injury group, two patients (9.1%) received an orthopedic operation (one for a hip luxation and one for a severe knee injury) and one patient a general surgery operation (4.5%) (emergency exploratory laparotomy due to hemodynamic instability).

In the upper cervical injury group, the rate of patients that received any type of spinal surgery was lower than in the lower cervical injury group (*p >* 0.05) and the thoracolumbar injury group (*p >* 0.05).

The rate of patients that received cranial surgery in this group was lower too (*p >* 0.05).

### ICU care

On arrival, 10/22 patients in the upper cervical injury group were transferred to ICU (45.5%); seven of these were intubated and eight had an associated injury other than the head or spine. The rate of ICU admission was similar to the lower cervical injury group (45.5% vs 40%, *p >* 0.05), while it was lower than in the thoracolumbar injury group (62.9%, *p >* 0.05). In ICU, one patient developed a pulmonary infection and another succumbed to sepsis. No other complications occurred among the remaining patients. Among the seven patients that were admitted to ICU and who required mechanical ventilation, three were never extubated and died (mean ventilator days = 20.6) and four were extubated after a mean of 11.8 days. The mean ICU stay was 20.5 days (4–57) in the upper cervical injury group, while it was 10.7 days (1–32) in the thoracolumbar injury group and 8.5 days (2–29) in the lower cervical injury group. ICU length of stay was significantly longer in the upper cervical injury group when compared to the non-upper cervical injury group (*p = *0.018). Patients that required ICU in the upper cervical injury group were older when compared to all other injury groups (i.e., thoracolumbar, lower cervical and non-upper cervical injury groups- 60:49:45:48, *p >* 0.05).

### Outcomes

In-hospital mortality was observed in four patients in the upper cervical injury group (18.2%); three deaths occurred in ICU and one on the ward. None of the patients died as a direct consequence of the upper cervical spinal trauma; one died from sepsis, one from acute respiratory failure, and two from the severity of the cranial trauma (while one of these had also suffered a hypoxic insult in the pre-hospital setting). No death was reported in the lower cervical injury or thoracolumbar injury groups (*p = *0.003).

Therefore, 18 of the 22 patients with cranial plus concomitant upper cervical spine injury survived (81.8%). Eleven of these 18 surviving patients were discharged directly home, without requiring an interim transfer to a rehabilitation facility. There was no significant difference in the mean GCS at discharge among any group. In the upper cervical injury group, more than half of the surviving patients made a good recovery (10/18; 55.5%) while 4/18 (22.2%) had a moderate disability according to the GOS At the time of discharge, ten patients had GOS 5, four had GOS 4, and four had GOS 3. The mean total hospital stay was 16.5 days, without any significant difference among the groups.

## Discussion

The rate of concomitant cranial and *upper cervical spine* injury was 1.4% in our series. Female gender and age ≥ 55 appeared to be risk factors for this pattern of injury. RAs were the most common mechanism of injury and all pedestrians’ injuries occurred in this group. Additionally, ICU stay and mortality rates were significantly higher in this group, even though no clear answer for this pattern could be defined.

A recent literature review with metanalysis found 11 publications reporting the prevalence of cervical spinal injuries in patients with TBI and prevalence ranged from 1.6 to 11.4% [2]. Nevertheless, it was possible in only two papers to retrieve the proportion of upper cervical injuries. In Holly et al.’s series, the rate of upper cervical injury was 2.5% (11/447), while in Tian et al.’s study, the most frequently injured level was the upper cervical spine and this was 3.6% (37/1026) [17, 19]. Both studies included only patients with moderate and severe TBI, and this could explain the lower incidence in our series of 1.4%, where we included complicated mild TBI too. This might also be explained by the previously reported higher incidence of cervical injuries in patients with lower GCS [13, 18, 21, 30]. However, such a correlation between low GCS and associated cervical injury was not confirmed in all the studies [15].

Differently from most of the previous series examining concomitant TBI and general cervical injury, we found a higher proportion of upper cervical injuries in females_._ Additionally, our patients were older than what has previously been reported [15, 17–19, 21]. Only one paper found a higher incidence of concomitant TBI and any cervical injury in older females like ours [30].

RAs represent the most common cause of spinal injury and TBI in many regions of the world. Nearly half of our patients with concomitant TBI and upper cervical injury suffered from this mechanism of trauma, a proportion not significantly different from the other examined groups [3, 31, 32]. Additionally, while stratifying for the exact mechanism of RA, we found no significant association between the mechanism of injury and upper cervical injury, despite what others found for general cervical injuries [21, 30]. An exception is represented by pedestrians: all pedestrians in our series of concomitant craniospinal injuries (admittedly only three) were in the group with an associated upper cervical injury, suggesting that this segment is particularly vulnerable in pedestrians struck by motor vehicles [33]. Holly et al. also found that two out of their 11 patients suffering from an upper cervical injury were pedestrians [17]. Other authors found no statistically significant difference in the incidence of TBI and associated general cervical injuries in pedestrians vs car accidents; whereas others instead found a higher incidence of cervical injuries in patients sustaining a motorcycle accident [15, 19].

Due to the strict anatomical and biomechanical relationship between the skull base and the upper cervical spine, one would expect a higher incidence of injuries in this area rather than in the skull vault, as found by others for concomitant TBI and general cervical trauma [19]. However, in our series, the rate of vault fractures was double that of skull base fractures. Again, we postulate that the high proportion of mild TBIs in our series could affect these data, as skull base fractures are often associated with severe head injury [34]. We attribute to the same reason the absence in our series of condyle fractures [35].

There might, however, be an underestimation, because we did search for brain trauma as a first selection parameter and then for concomitant spine trauma. It is unclear how many of the upper cervical spine trauma patients did experience mild TBI without intracranial hemorrhage (uncomplicated mild TBI) as we currently did not look for that actively. Although in daily life the rising incidence of upper cervical spine fractures in the elderly is seen with concomitant scalp wounds. A prospective study may look at that in detail.

Regarding the intracranial findings, we can only compare with Holly et al.’s series, which reported a high proportion of brain edema and intraventricular hemorrhage in patients with TBI and upper cervical injury^15^. In our series, none of our patients presented with these lesions as they are commonly encountered in severe TBIs, while subdural hematomas, subarachnoid hemorrhage, and epidural hematoma can be found ubiquitously in head-injured patients [36].

Once more, the absence of neurological deficit due to the C1–C2 injury in all our patients might be explained by the high rate of mild TBIs. It has, however, to be outlined that one of our upper cervical injury patients presented MRI signs of acute C-spine myelopathy due to an associated C6–C7 injury. However, the patient could not be clinically evaluated due to the severity of the associated injuries and the death that occurred thereafter.

Conversely, 8 out of 11 patients with upper cervical injury in Holly et al.’s series presented a neurological deficit by SCI [17]. Indeed, all the upper cervical injuries of our series were considered stable and did not require surgical management for stabilization or decompression. A limitation is that only 50% of our patients had an MRI; if clinical instability was not a concern after CT, an MRI was not always indicated as per institutional practice [23].

Selection bias is probably the reason for the absence of SCI, as most upper cervical spine fractures with SCI or vertebral artery lacerations die at the trauma scene.

Consistently with the other studies, a significant proportion of patients presented with injuries in body parts other than the head or spine [21]. However, in our series, none of the associated injuries was significantly associated with higher odds of upper cervical injury.

Even if it did not reach statistical significance, the total rate of associated injuries was inferior in the upper cervical injuries’ group when compared to the other groups. Again, although not significant, the rate of mild TBIs was higher in the upper cervical group. Due to the nature of our study, we could not establish in detail the energy of trauma but only how the injury occurred. Indeed, we cannot exclude that at least some of the RAs that constitute the majority of traumas in our series were indeed low-energy RAs. However, such findings could confirm a greater fragility of the upper cervical segment, as patients with an apparently milder injury suffered from such patterns of trauma. Actually, the literature about upper cervical injury (without TBI) agrees that this segment is particularly vulnerable to flexion–extension mechanisms of injury, especially in elderly patients. Degenerative changes and osteopenia may cause stiffness of the lower cervical spine which, in turn, might contribute to upper cervical spine injury even after low-energy trauma [37]. Older age could similarly explain the longer ICU length of stay of this group, even if the rate of overall ICU admissions did not differ between the groups [38, 39].

The high rate of mortality that was found in the upper cervical injury group is noted, especially in the absence of a high rate of severe and unstable cervical injuries. It is indeed well known that injuries in the upper cervical segment may cause inadequate diaphragmatic innervation and consequent respiratory dysfunction or apnea [17]. However, the mortality of our patients was 50% secondary to head injury, and 50% to general ICU complications.

The outcomes-at-discharge in the upper cervical injured group were in general satisfactory, and the registered number of disabled patients was attributable to the head or other injuries rather than to upper cervical injuries. Others reported much worse outcomes, but, again, their series included much more severely injured patients [17].

### Limitations

This was a single-centre retrospective study with its inherent limitations of selection bias and confounding. The small sample population undermined the power of the statistical analysis, both on univariate and multivariate analyses. In particular, a multivariate logistic regression model based on the variables that statistically performed better on univariate analysis showed no statistical significance. Additionally, due to the retrospective nature of the study, some granular information was not retrievable (i.e., components of the GCS or AIS head). However, the available results may constitute the initial evidences for future prospective studies recruiting a higher volume of cases. The baseline diagnosis upon which the search was conducted was traumatic brain injury, based on an abnormal CT head scan, and admission to the hospital. This, therefore, did not capture all mild head injuries, such as concussions, which may have had associated spinal injuries, but which were not admitted to the neurosurgical tertiary centre. Additionally, a converse analysis of patients with a spinal injury and a specific focus on the TBI population was not conducted as a spinal injury registry was not available. As per pragmatic network services, some head injuries were not serious enough to warrant transfer to the tertiary neurosurgery centre; concomitant craniospinal injuries of lower severity may have thus not been captured. On the other hand, the tertiary centre involved is the only such centre and serves a defined region and population. This provides reliable information for concomitant craniospinal injury among those who suffered a TBI which required specialized neurosurgical input, including clinicoradiological observation that may or may not have led to surgical intervention. It must be, however, recognized that the admission to a neurosurgical ward might be somehow prone to subjectivity of the on-call neurosurgeon and this could have been a bias regarding patient’s inclusion.

## Conclusion

The rate of concomitant cranial and *upper cervical spine* injury was 1.4%. Risk factors for this pattern included female gender, age ≥ 55, and pedestrians. There was an association between the upper cervical injured group and longer ICU stay as well as higher mortality rates, even though they could not be directly attributable to the upper cervical injury itself.

## Data Availability

The data that support the findings of this study are available from the 1st author, [NM], upon reasonable request.
